# Measles in Vaccinated People: Epidemiology and Challenges in Surveillance and Diagnosis in the Post-Elimination Phase. Spain, 2014–2020

**DOI:** 10.3390/v13101982

**Published:** 2021-10-02

**Authors:** Noemí López-Perea, Aurora Fernández-García, Juan Emilio Echevarría, Fernando de Ory, Mayte Pérez-Olmeda, Josefa Masa-Calles

**Affiliations:** 1 Centro Nacional de Epidemiología, Instituto de Salud Carlos III (ISCIII), Av. Monforte de Lemos 5, 28029 Madrid, Spain; nlopezp@isciii.es (N.L.-P.); jmasa@isciii.es (J.M.-C.); 2 CIBER de Epidemiología y Salud Pública (CIBERESP), Instituto de Salud Carlos III (ISCIII), Av. Monforte de Lemos 5, 28029 Madrid, Spain; jeecheva@isciii.es (J.E.E.); fernandodeorym@gmail.com (F.d.O.); 3 Centro Nacional de Microbiología, Instituto de Salud Carlos III (ISCIII), Ctra. Majadahonda-Pozuelo s/n, 28220 Madrid, Spain; mayteperez@isciii.es; 4 CIBER de Enfermedades Infecciosas, Instituto de Salud Carlos III (ISCIII), Av. Monforte de Lemos 5, 28029 Madrid, Spain

**Keywords:** measles, surveillance, elimination, vaccination, vaccine failure, waning immunity, nosocomial transmission, healthcare workers

## Abstract

The MMR vaccination program was introduced in Spain in 1981. Consistently high vaccination coverage has led to Spain being declared free of endemic measles transmission since 2014. A few imported and import-related cases were reported during the post-elimination phase (2014 to 2020), with very low incidence: three cases per million of inhabitants a year, 70% in adults. In the post-elimination phase an increasing proportion of measles appeared in two-dose vaccinated individuals (up to 14%), posing a challenge to surveillance and laboratory investigations. Severity and clinical presentation were milder among the vaccinated. The IgM response varied and the viral load decreased, making the virus more difficult to detect. A valid set of samples (serum, urine and throat swab) is strongly recommended for accurate case classification. One third of measles in fully vaccinated people was contracted in healthcare settings, mainly in doctors and nurses, consistent with the important role of high intensity exposure in measles breakthrough cases. Surveillance protocols and laboratory algorithms should be adapted in advanced elimination settings. Reinforcing the immunity of people working in high exposure environments, such as healthcare settings, and implementing additional infection control measures, such as masking and social distancing, are becoming crucial for the global aim of measles eradication.

## 1. Introduction

The World Health Organization for Europe declared measles eliminated in Spain in 2017, defined as the absence of endemic measles transmission for a period of at least 36 months. Since 2014, measles cases and outbreaks reported in the country have been imported or import-related [1,2].

As the measles virus continues to circulate in many areas of the world, including some of our neighbouring European countries, imported cases and outbreaks continue to be reported in Spain in the post-elimination phase [2].

As elimination progresses and high vaccination coverage is sustained, the epidemiology of measles evolves and presents a typical profile: low or very low incidence, small outbreak size, imported origin of infection and disease affecting mainly unvaccinated adults [3].

Much less frequently, but not in a negligible proportion, measles appears in vaccinated individuals, playing an important role in the epidemiology of the disease [4,5,6,7]. As vaccination coverage increases in the general population, a proportional increase in the frequency of measles cases among the vaccinated would be expected [4]. Detection of infection in vaccinated individuals poses a challenge to global and regional elimination efforts [8].

To sustain measles elimination, in addition to maintaining high coverage with two doses of measles, mumps and rubella (MMR) vaccine, sensitive surveillance and an effective outbreak response are needed. A first dose of MMR vaccination was introduced in the Spanish schedule for child vaccination in 1981; a second dose was included in 1996. Vaccination coverage for the first MMR dose has been above 95% since 1999, while for the second dose it is slightly below 95% [9].

The measles-containing vaccine (MCV) is highly effective, but a small number of two dose-vaccinated people (around 3%) [4,10] exposed to the virus contract measles. In Spain, from 2003 to 2014, around 3.5% of confirmed cases had received two doses of MMR vaccine [11], most of them having received the second dose more than 10 years prior [2].

A high intensity of exposure is known to play a role in the occurrence of measles breakthrough cases, mainly in previously vaccinated individuals [12,13]. Measles among vaccinated healthcare workers has been central in many outbreaks declared in healthcare settings in the post elimination era [6,14,15,16].

However, the role of measles-infected vaccinated people in transmission of the virus remains unclear [5,12,17,18].

In an individual with a history of vaccination, vaccine failure is classified as primary, depending on measles IgG avidity (primary vaccine failure—PVF; low avidity) or as secondary, due to waning of immunity (breakthrough cases; high avidity). In the case of PVF, individuals failed to develop a primary immune response following vaccination. Clinical presentation and laboratory evaluation in a case with PVF is no different from a primary measles infection in an unvaccinated individual [12]. However, the clinical presentation of measles breakthrough cases varies from a milder modified disease to an illness that meets the case definition. This milder presentation makes the identification of a case more difficult [12,18,19].

Laboratory investigations of suspected cases acquires a relevant function in highly-immunised populations, as current laboratory diagnosis algorithms pivoting on antibody response (IgM and IgG) could result in misclassified cases in low measles virus (MV) circulation settings [11].

Surveillance in the advanced elimination phase (i.e., highly immunised population) requires high sensitivity and specificity to identify and control any MV circulation; the positive and negative predictive values of the IgM assay results diminish. The best method to confirm these cases with an appropriately timed sample is RT-PCR [12]. For this reason, the recommendation is to sample appropriately timed specimens for molecular (PCR in throat swab/urine sample) and serological (measles specific IgM in serum sample) diagnosis. Other techniques can be used in order to raise diagnosis performance, such as measles-specific IgG (seroconversion) and detection of IgG avidity (low avidity confirms the case). Finally, genotyping is crucial for the classification of cases in recently vaccinated patients, as identification of the vaccine strain rules out cases [12].

This study describes the clinical, epidemiological and laboratory profile of measles in the post-elimination phase in Spain and explores the challenges posed by an increasing proportion of measles in vaccinated individuals on the measles surveillance system.

## 2. Materials and Methods

Measles surveillance in Spain is supported by the National Epidemiological Surveillance Network (Red Nacional de Vigilancia de España, RENAVE in Spanish). This network is in charge of national surveillance of infectious diseases and outbreaks, and is managed by the National Centre for Epidemiology (CNE in Spanish). Data related to measles cases were obtained from the RENAVE.

### 2.1. Case Definition

Case definitions used for measles surveillance in Spain fit the European Commission case definitions: “Possible cases” are those meeting the clinical criteria; “Probable cases” are those meeting the clinical criteria with an epidemiological link to a confirmed measles case; and a “Confirmed case” is an individual not recently vaccinated who meets clinical and laboratory criteria [18].

A more sensitive spectrum of clinical criteria is used in the post-elimination phase by epidemiologists in the field in order to include all suspicious cases. We have developed a new variable called “modified measles” for classifying those cases that do not match the classic clinical presentation (maculopapular rash, fever and any of the following three: cough, coryza, conjunctivitis [20]).

### 2.2. Variables

To characterize the cases, the following variables were considered: case classification, year of reporting, sex, age group (<1 year, 1–4 years, 5–19 years, ≥20 years), origin of infection (imported, import-related, endemic or unknown [20]), number of MMR vaccine doses received, time from the last MMR dose, clinical presentation (classic measles or modified measles), severity (hospitalisation), measles related to healthcare settings, and laboratory infection markers such as IgM and RT-PCR.

We describe the overall measles incidence rates in Spain in the post-elimination phase (2014–2020). To identify possible differences according to vaccination status, three comparison groups were defined: unvaccinated, vaccinated with one MMR dose and vaccinated with at least two doses. The latter group will also be named “fully vaccinated” throughout this manuscript. A vaccine dose was only considered valid if administered at least 21 days prior to the rash onset.

To calculate incidence rates, population information was obtained from the National Institute of Statistics (Instituto Nacional de Estadística-INE) [21].

A Fisher’s exact test was used to compare some selected variables between the three defined groups of comparison.

Excel 2010© and Stata 15.1© (1985–2017 StataCorp LLC. StataCorp. 4905 Lakeway Drive. College Station, TX 77845, USA) were used for the statistical analysis.

## 3. Results

In Spain during the post-elimination phase, 988 measles cases were reported to the RENAVE, with an overall incidence rate of 3.0 cases per million inhabitants (ranging from 0.8 cases/10^6^ in 2015 and 2016 to 6.1 cases/10^6^ inhabitants in 2019).

The distribution of measles cases in terms of sex was similar (502 male/486 female; 50.8% vs. 49.2%). Although fully vaccinated cases were more predominant in women (55.9%, 64/121), no significant differences were found (Fisher’s exact test *p* = 0.429).

By age group, 95 (9.6%) were infants (<1 year); 82 (8.3%) were 1–4 years old; 130 (13.2%) were 5–19 years old and 681 (68.9%) were 20 years or older. The incidence rate of measles in the post-elimination phase ranges widely by age: 33.9 cases/million inhabitants for younger than one year; 6.7/10^6^ for 1–4 years old; 2.63/10^6^ for 5–19 years old and 2.6/10^6^ inhabitants among those 20 years and older (Table 1).

According to the origin of infection, most of the cases were linked to a measles case imported from another country (95; 93.1%)—mainly from Europe (58/102; 69.0%). Italy, Ukraine and Romania accounted for 68.0% of imported cases.

Information about vaccination status was available for 863 cases (87.3% of all cases); 76.6% (661/863) of cases were not vaccinated, 9.4% (81/63) had one dose of MMR vaccine and 14.0% of cases (121/863) had received two doses or more (Table 1).

**Figure 1:**
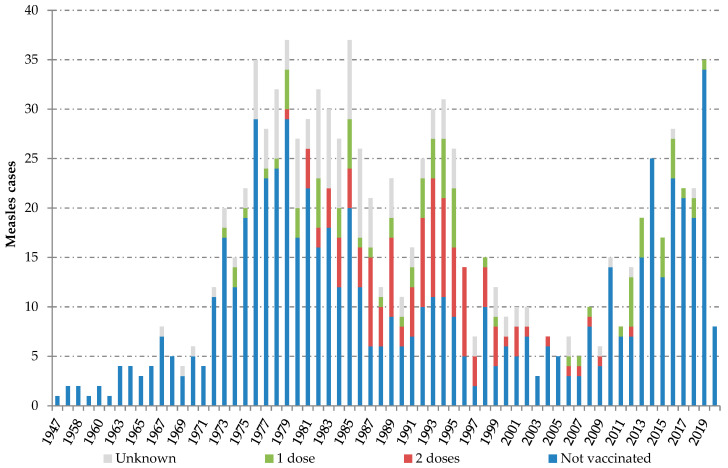
Measles cases by birth cohort and measles, mumps and rubella (MMR) vaccination status. Spain, 2014–2020. Source: National Epidemiological Surveillance Network, National Centre for Epidemiology, ISCIII.

Individuals with confirmed measles reported between 2014 and 2020 were born between 1947 and 2020. Two peaks can be observed along the series of cases classified by birth year. The “adult peak”, including measles infection in people born between 1973 and 1999, and the “children peak”, including measles among children born from 2010 onwards. Most fully vaccinated cases (81.0%; 98/121) belonged to the cohorts born between 1981 and 1996 (Figure 1).

For 165 cases, the date of the last MMR dose was available. The median elapsed time from the last dose to rash onset was 18.8 years for those with ≥2 doses and 16.2 for those with one dose (Table 2).

**Figure 2:**
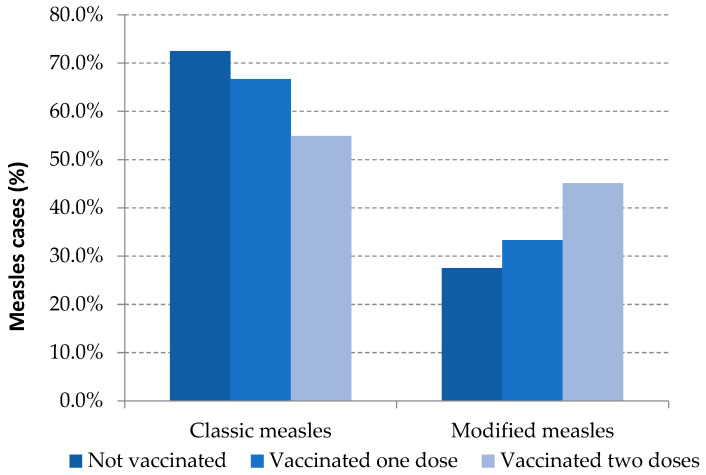
Measles clinical presentation and vaccination status. Spain, 2014–2020.Source: National Epidemiological Surveillance Network, National Centre for Epidemiology, ISCIII.

Modified measles was the clinical presentation in 31.2% of the total reported cases: 27.5% were unvaccinated cases, 33.3% were those with one dose of MMR and 49.6% were fully vaccinated cases. These differences were statistically significant between unvaccinated and vaccinated with two or more doses (Table 3, Figure 2).

Regarding the severity of the disease, the number of hospitalized cases was inversely proportional to the number of MMR doses received: 38.3% in unvaccinated, 24.7% among one dose-vaccinated and 9.4% in fully vaccinated; differences were statistically significant (Table 3).

The proportion of cases having acquired measles in a healthcare setting as workplace (HCS-WP) was 6.1% among unvaccinated cases, 9.9% in vaccinated with one dose and 27.3% in cases vaccinated with two or more doses. Differences found were significant between unvaccinated vs. fully vaccinated and between one-dose vaccinated vs. two-dose vaccinated (Table 3).

A total of 84 measles cases were reported in an HCS-WP; most of them (71; 84.5%) were healthcare workers (HCW): 32.1% nursing, 21.4% medical staff and 31.0% other HCW. 15.5% of the cases related to healthcare working environment occurred among administrative staff and other people working at healthcare settings (Table 4).

Among HCW, 40.8% were unvaccinated while 45.1% had received two doses or more. Just 9.9% of these workers had one dose of vaccine (Table 4). Non-HCW cases that were nonetheless working in healthcare environments, were mostly unvaccinated (84.6%) (Table 4).

Of the fully vaccinated cases, 26.4% (32/121) occurred among healthcare workers— mostly nursing staff (39.4%) and doctors (33.3%). Seven cases in this working group had one dose of MMR vaccine. Most cases had not received any dose of vaccine (41; 48.2%) (Table 4).

Most cases were laboratory confirmed (926, 93.7%), 52 (5.3%) were epidemiologically linked (probable cases) and 10 (1.0%) cases were clinically compatible (possible cases).

Regarding the laboratory study, for most cases at least one biological specimen was obtained (949/988; 96.1%). Of them, 55.1% had the desirable set of samples (throat swab /urine for PCR test, and serum sample for IgM serology). If only laboratory-confirmed cases are taken into consideration (925/988; 93.7%), 57.9% of cases had enough samples to perform both PCR and serology; 28.2% had only a throat swab/urine sample and 13.9% only had a serum sample for IgM serology. No differences in the set of clinical samples taken were found among groups depending on vaccination status (Table 5).

Regarding the laboratory tests on the basis of which cases were confirmed, 40.1% of cases were confirmed by both positive IgM and positive PCR; 38.8% only by a positive PCR result and 21.1% only by a positive IgM result (Table 6).

Reducing the analysis to the 536 laboratory-confirmed cases with a valid set of clinical samples available for testing, 81.3% of cases had a positive IgM test; 86.9% had a PCR positive test. The proportion of cases with a positive IgM test result among those with two doses of vaccine fell to 43.5%. Statistical differences were found among groups of comparisons based on vaccination status (Table 6).

## 4. Discussion

Achieving elimination of measles relies on a sustainable, accessible and successful vaccination programme maintained over time. The MMR vaccine programme started in Spain in 1981 and has had an excellent acceptance rate by the population since then. The high level of vaccination coverage achieved among infants and children over the decades has led to Spain being declared free of endemic transmission of measles since 2014 [1].

In the post-elimination phase (2014–2020), measles in Spain presents the profile of the disease in advance elimination settings: a very low incidence (three cases per million inhabitants), the origin of infection is imported or import-related, and most cases occur in adults, while the disease remains extremely infrequent among children and infants.

Generally, individuals with confirmed measles infection reported in the post-elimination phase were born between 1947 and 2020 and 78.4% were people born in Spain. Individuals having received two doses were born in or after 1981, with a higher proportion—86.8%—born in Spain. Moreover, measles is increasingly appearing in fully vaccinated persons: 14.0% of total confirmed cases in Spain for the period 2014–2020, while this proportion was around 3.0% in the previous ten years [7] when endemic transmission of MV with moderate size outbreaks was still reported in the country.

Most fully vaccinated cases infected with measles belonged to the cohorts born between 1981 and 1996. This result matches the conclusions of the second national seroprevalence study in Spain [22] performed in 2017. This study suggests a decline in the population with protective measles antibody titers for the cohorts born between 1978 and 1997 compared to those born before 1978, whose proportion of protected population is above 95%. The proportion of people protected ranges from 91.5% for those born from 1978 to 1987, to 86.9% for those born between 1988 and 1999. The lowest proportion of individuals with protective antibody titers corresponds to the cohorts where all the individuals were targeted for receiving two doses of MMR vaccine. A combination of the waning of vaccination immunity together with the dramatic decline of measles circulation in Spain since the decade of 1990 [9], which hinders exposure to natural boosters, could explain the prevalence of measles in individuals previously vaccinated with two doses among adults belonging to these cohorts.

The median of years elapsed between the last measles vaccination and the rash onset in fully vaccinated persons increased as we move further away from the start of the vaccination programme. The elapsed period was 4 years for cases which occurred in 2003–2014 [11] vs. 18.8 years elapsed for cases reported in 2014–2020. This increase over time in the number of years between the date of the last MMR dose and rash onset reflects the fact that the vaccination programme has been working well in Spain for many years.

Measles in previously vaccinated individuals presents some differential features in clinical and laboratory characterisations. It is well known that a modified mild illness, sometimes unrecognizable, is frequent in vaccinated people [16,19]. In our study, up to one third of total measles cases and half of those occurring in fully vaccinated persons could have been undetected because of the lack of the classical set of symptoms triggering surveillance activities.

Introducing changes in the case definition of a reportable infectious disease in the European Union (EU) may be delayed because it needs the approval of the European Commission. Nevertheless, following the proposal made by Portugal [16] to expand the EU measles case definition in order to increase sensitivity in detecting cases among vaccinated people, some changes were introduced in the metadata for reporting measles cases to the ECDC. In this regard, for those cases confirmed with two doses of MMR vaccine, it is necessary to indicate whether they meet the clinical criteria of a measles case.

One third of measles patients needed to be hospitalized because of the severity of the illness presentation. In our study, the odds of hospitalization decreased with the number of MMR doses received, showing evidence of protection conferred by vaccination against severe measles, as demonstrated in other studies [11].

HCS-WP has proven to be an effective environment for spreading airborne infectious diseases. For some highly controlled infectious diseases, such as measles in post-elimination settings, the healthcare environment might act as a reservoir of infectious agents while agent circulation is extremely low in the general population [6,15,16]. A prolonged high-intensity exposure to an acute case in a medical setting is thought to play an important role in the occurrence of measles in vaccinated individuals [8,12,17,18].

Measles reported in HCS-WP occurred both in HCW and health science students, and in those who perform their work in a healthcare facility (e.g., administrative staff and others). The greatest number of measles cases was reported among nursing staff and resident doctors, who are usually in closer contact with patients. Interestingly, around 40% of HCW having measles were unvaccinated. This calls for a review of the vaccination protocols for staff working in healthcare settings.

Around 10% of measles cases confirmed in the post-elimination phase in Spain were infected while working in a healthcare setting; this proportion rises to 27% among cases having received two doses of MMR vaccine. Some factors may contribute to the fact that measles acquired in HCS-WP is overrepresented among fully vaccinated cases. A high proportion of current working-age people are adults likely to have received two doses of MMR vaccines (by childhood vaccination and/or by tailored vaccination programs for HCW) between 20 and 30 years before the exposure occurred. In this regard, protocols for ensuring measles immunity of HCW should also include how to take action in HCW that have already received two doses of MMR vaccine [23].

Explosive outbreaks affecting vaccinated children exposed to super-spreader measles cases has been published [13], but in our study they have not played a major role in the spread of measles.

Laboratory confirmation of suspected cases of measles becomes more challenging in post-elimination countries. The collection of appropriate specimens for serology and PCR may be more difficult in settings where vaccine coverage is high, and a rash illness in vaccinated individuals who are presumptively immune is not often suspected as being measles [12]. Moreover, implementation of control measures and a rapid laboratory confirmation are critical for reducing secondary exposure and transmission and strongly recommended for accurate case classification [12,20].

Because of the lack of reliability of IgM for confirming or ruling out measles infection in a highly immunised population with a very low prevalence of disease, identifying the RNA virus in throat swab/urine is the best method for confirmation. Following this principle, the WHO proposes performing PCR as the first test in laboratory algorithms for testing suspected measles in elimination settings [12]. If only a negative PCR result is available, measles cannot be ruled out. When the PCR result is negative or inconclusive, additional immunoassays (IgG in acute-convalescent paired samples, IgG avidity or neutralizing antibodies detection) should be performed in order to confirm or discard the case [8,12].

In Spain, since the measles elimination plan was established in 2001, adequate clinical samples for serology tests and PCR were taken, on average, for half of the reported suspicious clinical cases [2,24]. In the post-elimination phase, this proportion is maintained, and around 58% of cases were confirmed using both detection of specific IgM, and PCR tests; the rest of the measles infections were confirmed by a single test due to the lack of appropriate specimen collection, showing great room for improvement.

Our laboratory surveillance data show that there are no differences in the set of clinical samples taken among groups depending on the vaccination status. The high MMR population coverage sustained in Spain for decades may explain the awareness by epidemiologists and healthcare providers of measles in vaccinated people.

According to our results, and focusing on cases confirmed by detection of specific IgM and PCR, up to 14.5% of confirmed cases in the fully vaccinated could be misdiagnosed if a single sample is taken. Strikingly, this proportion rises to 56% if throat swab/urine samples, in addition to serum samples, were not collected. To cope with the added difficulty of confirming measles cases in post-elimination settings, both types of specimens must be collected [12,20].

The current guidelines for measles surveillance [20] together with the Spanish Plan for Measles and Rubella Elimination [25] need to be updated shortly in Spain. The clinical and laboratory features of measles breakthrough cases should be included in case definitions and in laboratory algorithms to diagnose measles, according to the specific guidelines for countries close to elimination provided in the latest laboratory protocols of the WHO [12]. In addition, the new protocols should pay special attention to minimizing measles transmission in healthcare settings [26].

Additional technical and human resources have to be allocated to National and Regional Reference Laboratories for Measles Surveillance. It is also necessary to strengthen those facilities dedicated to surveillance and public health response, to contribute to the progress of elimination of measles in Europe and measles eradication globally.

The COVID-19 experience leading to expansion of the recommendations for using non pharmaceutical interventions (e.g., masks and social distancing) to minimise transmission of airborne infectious disease, such as measles, in emergency rooms and other public areas shared by patients, workers and the public, should be incorporated.

The increasing presence of measles cases among properly vaccinated individuals, as expected in Spain at this moment, may be misinterpreted by the public as vaccine ineffectiveness. In this regard, public health authorities should make efforts to ensure that the information received by the public and the media is appropriate and accurate [4].

The goal of measles elimination is not threatened by measles in vaccinated individuals [5,6,12,27], but they will play an important role in managing outbreaks in the near future [28].

## 5. Conclusions

Spain faces a measles post-elimination phase; the disease presents the profile of measles infection in a highly immunised population, with an increasing presence of measles among vaccinated individuals. Surveillance protocols and laboratory technical resources should be adapted to clinical, epidemiological and immunological features of measles in advanced elimination settings for detecting and controlling any circulation of the virus in the population.

Intense exposure to individuals with contagious measles, as occurs in HCS-WP, plays an important role in re-infection of vaccinated people. National protocols aiming to both minimize measles transmission in healthcare environments and to ensure measles immunity in people working in HCS-WP need to be updated.

## Figures and Tables

**Table 1:** Measles cases with known vaccination status by age group and number of MMR * vaccines. Spain, 2014–2020.

		Vaccination Status							
Age Group	IR **	Unvaccinated		1 Dose		≥ 2 Doses		Total	
		*n*	%	*n*	%	*n*	%	*n*	%
**<1 year**	33.0	93	0.1%	1	1.1%	-	-	94	10.9%
**1–4 years**	6.7	61	0.1%	20	24.7%	-	-	81	9.4%
**5–19 years**	2.6	91	0.1%	7	6.0%	18	13.8%	116	13.4%
**≥20 years**	2.6	416	0.6%	53	9.3%	103	15.1%	572	66.3%
**Total**	3.0	661	75.6%	81	9.4%	121	14.0%	863	100%

* Measles, mumps and rubella vaccine (MMR). ** Incidence rate: cases/10^6^ inhabitants. Source: National Epidemiological Surveillance Network, National Centre for Epidemiology, ISCIII.

**Table 2:** Time (years) since last measles vaccination to rash onset date in 165 measles cases. Spain, 2014–2020.

No. of Vaccine Doses	No. of Cases	No. of Cases with Dates for Vaccination	Time (Years) from Vaccine to Rash		
			Mean *	Median *	Range
**≥2 doses**	121	102	18.0	18.8	1.0–31.5
**1 dose**	81	63	16.2	18.2	1.0–43.2

* Years since last vaccination. Source: National Epidemiological Surveillance Network, National Centre for Epidemiology, ISCIII.

**Table 3:** Global characteristics for measles cases. Spain, 2014–2020.

	Unvaccinated		1 MCV Doses		≥2 MCV Doses		Total		*p* Value *		
	*n*	%	*n*	%	*n*	%			0 vs. 1 Doses	0 vs. 2 Doses	1 vs. 2 Doses
**Clinical presentation**											
**Classic measles**	479	72.5%	54	66.7%	61	50.4%	594	68.8%	0.295	0.000	0.029
**Modified measles**	182	27.5%	27	33.3%	60	49.6%	269	31.2%			
**Hospitalization**											
**Yes**	253	38.3%	20	24.7%	12	9.9%	285	33.0%	0.020	0.000	0.006
**No**	408	61.7%	61	75.3%	109	90.1%	578	67.0%			
**Healthcare working environment**											
**Yes**	40	6.1%	8	9.9%	33	27.3%	81	9.4%	0.226	0.000	0.002
**No**	621	93.9%	73	90.1%	88	72.7%	782	90.6%			

* Fisher’s exact test. Source: National Epidemiological Surveillance Network, National Centre for Epidemiology, ISCIII.

**Table 4:** Measles cases reported in healthcare settings, by professional group and vaccination status. Spain, 2014–2020.

Healthcare Workers (HCW)									
		*n*	%	Unvaccinated (*n*, %)		1 MCV Doses (*n*, %)		≥2 MCV Doses (*n*, %)	
**Nursing**	**Registered nurses**	19	26.8%	6	20.7%	1	14.3%	11	34.4%
	**Auxiliary nurses**	8	11.3%	3	10.3%	3	42.9%	2	6.3%
**Medicine**	**Medical officers**	6	8.5%	2	6.9%	-	-	3	9.4%
	**Resident doctors**	12	16.9%	3	10.3%	1	14.3%	8	25.0%
**Health Science student**		5	7.0%	3	10.3%	-	-	2	6.3%
**Orderly**		8	11.3%	5	17.2%	1	14.3%	2	6.3%
Other HCW *		13	18.3%	7	24.1%	1	14.3%	4	12.5%
**Total**		71	100%	29	40.8%	7	9.9%	32	45.1%
									
**No Healthcare Workers (HCW)**									
		*n*	%	**Unvaccinated (*n*, %)**		**1 MCV doses (*n*, %)**		**≥2 MCV doses (*n*, %)**	
**Administrative staff**		7	53.8%	7	63.6%	-	-	-	-
**Other workers in healthcare settings**		6	46.2%	4	36.4%	1	100%	1	100%
**Total**		13	100%	11	84.6%	1	7.7%	1	7.7%

* Other HCW: Physiotherapist, Rx Technician, dentistry, unspecified HCW. Source: National Epidemiological Surveillance Network, National Centre for Epidemiology, ISCIII.

**Table 5:** Laboratory study of laboratory-confirmed measles cases. Spain, 2014–2020.

Sample	Global		Unvaccinated		1 Dose		≥2 Doses		*p* Value *		
	*n*	%	*n*	%	*n*	%	*n*	%	0 vs. 1 Doses	0 vs. 2 Doses	1 vs. 2 Doses
TS **/Urine + Serology	536	57.9%	344	55.5%	50	65.8%	62	55.9%	0.098	0.844	0.245
**TS/Urine (only)**	260	28.1%	191	30.8%	19	25.0%	36	32.4%	0.159	0.514	0.529
**Serology (only)**	129	13.9%	85	13.7%	7	9.2%	13	11.7%	0.740	0.355	0.328
**Total**	925	100%	620	100%	76	100%	111	100%			

* Fisher’s exact test. ** Throat swab. Source: National Epidemiological Surveillance Network, National Centre for Microbiology, National Centre for Epidemiology, ISCIII.

**Table 6:** Laboratory performance for measles cases with both samples (serum and throat swab/urine). Spain, 2014–2020.

	Global		Unvaccinated		1 Dose		>2 Doses		*p* Value *		
	*n*	% **	*n*	%	*n*	%	*n*	%	0 vs. 1 Doses	0 vs. 2 Doses	1 vs. 2 Doses
**IgM +**	436	81.3%	302	87.8%	38	76.0%	27	43.5%	0.027	0.000	0.001
**PCR+**	466	86.9%	314	91.3%	39	78.0%	53	85.5%	0.010	0.162	0.331

* Fisher’s exact test. ** % of positivity. Source: National Epidemiological Surveillance Network, National Centre for Microbiology, National Centre for Epidemiology, ISCIII.
